# Correlation between α-ketoglutarate and the oxidative stress response in patients with coronary heart disease and prognostic analysis of interventional therapy for coronary heart disease

**DOI:** 10.3389/fcvm.2025.1544537

**Published:** 2025-07-21

**Authors:** Huantang Deng, Yu Zhang

**Affiliations:** Department of Cardiology, Huizhou Central People's Hospital, Huizhou, Guangdong, China

**Keywords:** coronary heart disease, oxidative stress, interventional therapy, α-ketoglutarate, prognosis assessment

## Abstract

**Objective:**

To explore the correlation between α-ketoglutarate and the oxidative stress response in patients with coronary heart disease (CHD) and to analyze the prognosis of CHD patients receiving interventional treatment.

**Methods:**

A total of 318 CHD patients admitted from September 2020 to September 2023 were selected and divided into a conservative medical treatment group (159 patients) and an interventional treatment group (159 patients) according to the treatment plan. The interventional treatment group was divided into an event group and a nonevent group according to whether the primary endpoint event occurred; another 59 patients with healthy physical examination results during the same period composed the control group.

**The results:**

The serum TAC, SOD, CAT, and GSH levels in the conservative medical treatment group were lower than those in the control group (*P* < 0.05), and the serum MDA level was higher than that in the control group (*P* < 0.05). α-Ketoglutarate was correlated with oxidative stress indicators (TAC, SOD, CAT, and GSH) in patients with CHD (*P* < 0.05). A comparison of the α-ketoglutarate levels revealed that the control group > interventional treatment group > medical conservative treatment group (*P* < 0.05) and that the α-ketoglutarate level in the event group was lower than that in the nonevent group (*P* < 0.05). The ROC curve analysis results revealed that the area under the ROC curve (AUC) of α-ketoglutarate in the event group after interventional treatment was 702; the AUC of α-ketoglutarate in the no-event group was 802.

**Conclusion:**

α-Ketoglutarate is related to oxidative stress in patients with CHD. The lower the serum α-ketoglutarate level is, the greater the likelihood that adverse events will occur.

## 1 Introduction

In the past few decades, with the continuous development of the social economy, changes in people's lifestyles, and the continued prevalence of metabolic risk factors, the number of patients with hypertension, diabetes, and dyslipidemia has increased significantly in China. Most of the risk factors are hidden, and often, the blood vessels have already been damaged when discovered, and some have even led to serious adverse events such as myocardial infarction and stroke. Therefore, an increasing number of people need to take medication for life-long treatment, and the burden of CVD continues to increase. Cardiovascular disease has high morbidity and mortality rates and has become a major public health problem in both developing and developed countries. The prevention, early diagnosis, treatment decision-making, and prognosis improvement of such diseases have become hot topics of great concern in various clinical studies (1).

Cardiovascular diseases include coronary heart disease (CHD), congenital heart disease, cardiomyopathy, heart failure, etc. The incidence and mortality rates have always been at the top of the list in China. According to statistics, in 2019, cardiovascular diseases accounted for 40% of the causes of human death among rural and urban residents. Moreover, 2 out of every 5 deaths are due to cardiovascular disease, which places a considerable burden on humans and society. The impact of early preventive diagnosis and improved prognosis on the lifetime risk of CHD and the improvement of public health measures are crucial. Epidemiological studies have shown that risk factors such as smoking, inflammation, diabetes, hypertension, and genetic variation are involved in the pathophysiological progression of CHD. Lifestyle changes and genetic environmental factors are also based on traditional cardiovascular risk factors, such as an unbalanced diet, lack of physical exercise, etc. More than 80% of sudden cardiac deaths worldwide are caused by CHD, and the remaining 20% are caused by cardiomyopathy, congenital heart disease, ventricular hypertrophy, heart valve disease, and other heart diseases. CHD is caused by arterial stenosis caused by atherosclerosis. Arterial lumen stenosis in patients with CHD is related mainly to the occurrence of atherosclerotic plaques. The accumulation of lipid metabolism factors and increased levels of inflammatory factors and cell adhesion molecules aggravate the formation of atherosclerotic plaques. The formation of atherosclerotic plaques leads to endothelial dysfunction, which plays a crucial role in the pathogenesis of CHD (2). The current diagnosis of CHD is based on clinical symptoms, invasive angiography, or cardiac physiological stress testing. However, because these tests are susceptible to clinical bias, variable specificity, and sensitivity, diagnostic uncertainty and diagnostic delays may occur. Therefore, although we have made much progress in the diagnosis and prognosis prediction of CHD, new candidate biomarkers are still needed to diagnose and predict CHD (3).

α-Ketoglutarate acid is also called 2-oxoglutaric acid. It is a key intermediate metabolite in the tricarboxylic acid cycle and an important precursor for biosynthesis. It is highly evolutionarily conserved and ubiquitous in various biological systems in nature (such as microorganisms, plants, animals, and humans) (4). α-Ketoglutarate is at the center of the tricarboxylic acid cycle. A key step in the tricarboxylic acid cycle is the conversion of isocitric acid into α-ketoglutarate and then into hyaluronic acid. The tricarboxylic acid cycle not only provides energy by producing ATP through the catabolic pathway but also provides precursors for the biosynthesis of some substances. α-Ketoglutarate is the precursor for glutamic acid synthesis in the body. An important role of α-ketoglutarate is to complement the triacid cycle through the glutamine pathway (5). Under conditions of hypoxia or impaired mitochondrial function, α-ketoglutarate derived from the glutamine pathway is particularly important. It is an important raw material for body metabolism (6). In recent years, many studies have confirmed that the α-ketoglutarate pathway plays a pivotal role in tumor metabolism and hypoxic metabolism and is expected to become a new target for tumor metabolism therapy (7). In addition, α-ketoglutarate is often used as an ingredient in sports nutrition drinks, and when combined with arginine, it can quickly help athletes replenish their energy (8). Some studies have shown that in pulmonary surgery, the oral administration of α-ketoglutarate before surgery can not only improve a patient's postoperative pulmonary motor function but also reduce oxidative stress (9). α-Ketoglutarate can also promote cell energy metabolism, improve blood vessel elasticity, slow the aging of the body, and inhibit the occurrence and development of atherosclerotic lesions (10). However, there is a lack of corresponding evidence on the relationship between circulating α-ketoglutarate levels and prognosis in patients with CHD.

Recent studies have shown that oxidative stress caused by oxygen-free radicals plays an important role in the occurrence and development of coronary disease (11). Oxidative stress causes lipid peroxidation, oxidative damage to endothelial cells, the foaming of giant cells, the migration and proliferation of smooth muscle cells, etc., which are key links in the occurrence and development of atherosclerosis (12). The indicators that reflect the body's oxidative stress state mainly include the following two aspects. On the one hand, there are indicators of peroxidation. Their levels indirectly reflect the severity of human cells when they are attacked by free radicals, such as reactive oxygen species (ROS), oxidized low-density lipoprotein (OX-LDL), lipid peroxidation (LPO), and malondase (MDA); on the other hand, there are antioxidant indicators. Their level indirectly reflects the body's ability to scavenge oxygen free radicals, such as superoxide dismutase (SOD), glutathione, total antioxidant capacity, and glutamine peroxidation. The role of oxidative stress in atherosclerotic disease has been increasingly recognized.

Percutaneous coronary intervention (PCI) is a new method for treating CHD that has been developed in the past 30 years and does not require surgical thoracotomy or general anesthesia. The doctor punctures the artery (arm or leg) through the skin and uses catheters and other equipment under x-ray to treat stenosis or occlusion of the coronary artery to restore the lumen of the blood vessel and reopen the blood flow (13). However, owing to the different conditions of patients and the influence of many factors, such as changes in disease conditions, interventional therapy, as an invasive treatment method, still has risks. Therefore, this study aimed to explore the correlation between α-ketoglutarate and the oxidative stress response in patients with CHD and the value of α-ketoglutarate in evaluating the prognosis of CHD patients receiving interventional therapy.

## 2 Methods and materials

### 2.1 Basic information

A total of 318 patients with CHD admitted to our hospital from September 2020 to September 2023 were selected. Group assignment was determined according to the following clinical guidelines for CHD management (14): (1) coronary angiography results (SYNTAX score ≤22 for the interventional group vs. ≤32 for the conservative group); (2) symptom severity (CCS class I–II vs. III–IV); (3) ischemic burden on stress testing; and (4) shared decision-making with patients after comprehensive risk‒benefit evaluation. According to the treatment plan, the patients were divided into a conservative medical treatment group and an interventional treatment group, with 159 patients in each group. Another 59 healthy individuals who underwent physical examination during the same period composed the control group. Control subjects met all the following criteria: (1) no history of cardiovascular or cerebrovascular disease; (2) absence of subclinical atherosclerosis confirmed by carotid ultrasound (intima-media thickness <0.9 mm with no plaques); (3) normal blood pressure (<140/90 mmHg) without antihypertensive medication; (4) normolipidemia (total cholesterol <6.2 mmol/L, LDL-C < 4.1 mmol/L); (5) no diabetes (fasting glucose <7.0 mmol/L); and (6) no systemic inflammatory or metabolic disorders. The control group was rigorously matched to the CHD patient groups for age, sex, smoking history, body mass index (BMI), and lipid profiles (total cholesterol, LDL-C, HDL-C, and triglycerides) to minimize confounding bias. There was no statistically significant difference in sex, age, hypertension, or diabetes history among the three groups (*P* > 0.05) (Table 1**)**. This study was approved by the Institutional Review Board of Huizhou Municipal Central Hospital (Approval No. Ky112022005) and conducted in accordance with the Declaration of Helsinki. Written informed consent was obtained from all participants prior to enrollment.

**Table 1:** Comparison of general information among the three groups.

Variable	Medical conservative (*n* = 159)	Interventional therapy (*n* = 159)	Control group (*n* = 59)	Statistical value	*P* value
Gender (M/F)	105/44	103/46	38/21	*χ*^2^ = 0.731	0.684
Age (years)	58.20 ± 9.02	58.62 ± 8.85	59.05 ± 6.30	*F* = 0.568	0.579
Hypertension (*n*)	53	50	0	*χ*^2^ = 0.129	0.719
Diabetes (*n*)	18	25	0	*χ*^2^ = 1.318	0.251
Smoking (*n*, %)	45 (28.3%)	48 (30.2%)	15 (25.4%)	*χ*^2^ = 0.85	0.654
BMI (kg/m^2^)	25.8 ± 3.1	25.7 ± 2.9	24.9 ± 2.7	*F* = 0.92	0.401
Total Cholesterol (mmol/L)	4.9 ± 0.8	4.8 ± 0.9	4.7 ± 0.7	*F* = 0.75	0.475
LDL-C (mmol/L)	3.0 ± 0.7	2.9 ± 0.6	2.8 ± 0.5	*F* = 0.88	0.418
HDL-C (mmol/L)	1.2 ± 0.3	1.1 ± 0.2	1.3 ± 0.3	*F* = 1.12	0.329
Triglycerides (mmol/L)	1.6 ± 0.4	1.7 ± 0.5	1.4 ± 0.3	*F* = 1.05	0.354

Data are presented as the means ± standard deviations for continuous variables (age, BMI, lipid profiles) and *n* (%) for categorical variables (gender, hypertension, diabetes, smoking). Statistical comparisons: Chi-square test for categorical variables; ANOVA for continuous variables. BMI, body mass index; LDL-C, low-density lipoprotein cholesterol; HDL-C, high-density lipoprotein cholesterol.

### 2.2 Inclusion and exclusion criteria

The inclusion criteria were as follows: (a) met the diagnostic criteria for CHD and had at least one coronary artery segment lumen diameter ≥50% stenosis on coronary angiography records; (b) had an expected survival period >3 months; (c) were aged 40–75 years; (d) had good compliance and cooperation with the diagnosis and treatment; and (e) were informed about this study and signed the informed consent form.

The exclusion criteria were as follows: (a) severe liver or kidney dysfunction; (b) acute myocarditis, respiratory failure, or valvular heart disease; (c) frequent arrhythmia; (d) systemic immune system diseases or blood system diseases; and (e) pregnancy or lactation.

### 2.3 Methods

Fasting peripheral venous blood was collected from the conservative treatment group and the control group and was subsequently centrifuged, after which the serum was collected. Fasting peripheral venous blood (5 ml) was collected in EDTA-coated tubes and immediately placed on ice. The samples were subsequently centrifuged at 4 °C and 3000 rpm (1,500×*g*) for 15 min within 30 min of collection. Serum aliquots were stored at −80 °C until analysis (<3 months). For analysis, the samples were subjected to a single freeze‒thaw cycle and protein precipitation using ice-cold methanol (1:4 v/v; vortexed for 1 min, incubated for 10 min at −20 °C, and centrifuged at 14,000×*g* for 15 min at 4 °C). The supernatant was transferred for LC‒MS/MS or ELISA analysis. The enzyme-linked immunosorbent assay (ELISA) method was used to detect oxidative stress indicators, including total catalase (CAT), antioxidant capacity (TAC), malondialdehyde (MDA), superoxide dismutase (SOD), and glutathione (GSH), and the specific procedure was carried out strictly according to the instructions of the kit. Fasting venous blood samples were collected from the conservative treatment, control, and interventional treatment groups at the Department of Internal Medicine and centrifuged within 2 h (1,500×*g*, 15 min). After centrifugation, the supernatant was collected for later use. Serum α-ketoglutarate levels were determined via hydrophilic interaction liquid chromatography-tandem mass spectrometry (HILIC-LC/MS/MS; Shimadzu LCMS-8060 system). Chromatographic separation was performed with an ACQUITY UPLC® BEH Amide column (2.1 mm × 100 mm, 1.7 μm; Waters) with mobile phase A: 10 mM ammonium formate (pH 3.0) in water/acetonitrile (15:85, v/v) and mobile phase B: 10 mM ammonium formate in acetonitrile/water (90:10, v/v). Gradient elution: 0–2 min at 95% B; 2–4 min at 70% B; 4–6 min at 95% B. MS parameters: ESI−mode; MRM transition m/z 145.0 to 101.0 (collision energy: −15 eV); desolvation line temperature: 250 °C; nebulizing gas flow: 3 L/min. The oxidative stress indicators and α-ketoglutarate levels in the three groups were compared between the conservative medical treatment group and the control group; patients in the CHD interventional treatment group were followed up for 6 months, and 31 patients were ultimately confirmed to have the primary endpoint event (all-cause death or rehospitalization due to CHD). The interventional treatment group was divided into an event group and a nonevent group according to whether the primary endpoint event occurred, and the serum α-ketoglutarate level was measured. Primary endpoint events were strictly defined as follows: (1) all-cause mortality, verified through death certificates and hospital records; (2) CHD-related rehospitalization, requiring ≥24-h hospitalization with either (a) recurrent angina accompanied by new ischemic ECG changes and/or troponin elevation exceeding the 99th percentile upper reference limit (URL); or (b) unscheduled repeat revascularization (PCI/CABG) for documented lesion progression, excluding scheduled staged procedures and noncardiac admissions. All potential events were independently adjudicated by two cardiologists in a blinded manner, with discrepancies resolved by a third arbitrator. The 6-month follow-up protocol was initiated with baseline discharge education on symptom reporting, followed by structured monthly telephone interviews via validated questionnaires supplemented by scheduled clinic visits at 3 and 6 months (including ECG and laboratory testing). Electronic health record surveillance tracked unscheduled visits, with final endpoint confirmation conducted through a comprehensive medical record audit at study completion. The receiver operating characteristic (ROC) curve was used to evaluate the value of α-ketoglutarate levels in evaluating the prognosis of patients undergoing interventional treatment for CHD.

### 2.4 Interventional treatment methods

Percutaneous coronary intervention was performed. The specific operation method was as follows: Three days before surgery, the patient was treated with a combination of aspirin and clopidogrel. During the operation, coronary intervention was performed via the radial artery. The patient was placed in a supine position, with the right upper limb spread out 30°. The proximal and superior ends of the radial styloid process were removed, a puncture was made at the site of the strongest pulsation of the radial artery, and local anesthesia was applied. After the puncture is successful, a 5F arterial sheath can be inserted, a 100 U/kg heparin sodium injection [Sanofi (Beijing) Pharmaceutical Co., Ltd., national drug approval number J20180035, specification: 0.4 ml: 4,000 AxaIU] can be injected for coronary angiography, the sheath can be removed, and a radial artery compressor can be used to stop bleeding. After surgery, the patient was given a subcutaneous injection of 5,000 U low-molecular-weight heparin sodium once a day for continuous treatment for 1 week, and aspirin and clopidogrel were continued.

### 2.5 Sample size justification

*A priori* sample size estimation was performed via G*Power 3.1 software. On the basis of pilot data (*n* = 20 per group) showing mean α-ketoglutarate levels of 5.8 ± 0.7 μmol/L in nonevent patients vs. 4.6 ± 0.9 μmol/L in event patients, we calculated an effect size (Cohen's *d*) of 1.58. To detect this difference with 90% power at *α* = 0.05 (two-tailed), 11 participants per group were needed. Accounting for 20% potential attrition and subgroup stratification, we targeted ≥100 patients per treatment arm. For the event subgroup analysis, our observed effect size (*d* = 7.67; mean difference = 1.15 μmol/L, pooled SD = 0.15) yielded >99.9% *post hoc* power despite *n* = 31, exceeding conventional thresholds.

### 2.6 Statistical methods

Statistical analyses were performed via SPSS 26.0 (IBM Corp., Armonk, NY, USA). Continuous variables are expressed as the means ± standard deviations with 95% confidence intervals (CIs), whereas categorical data are presented as frequencies [*n* (%)]. Intergroup comparisons were performed as follows: (1) independent *t* tests with Welch's correction for two-group analyses (with Levene's test for homogeneity of variance); (2) one-way ANOVA with Tukey's *post hoc* test for multigroup comparisons; and (3) ANCOVA to adjust for age, hypertension, and diabetes where appropriate. Pearson correlations included Bonferroni correction for multiple comparisons (adjusted *α* = 0.01). To evaluate the independent prognostic value of α-ketoglutarate, multivariable Cox proportional hazards regression models were constructed: Model 1 was adjusted for age, diabetes history, and hypertension status; Model 2 was additionally adjusted for BMI, smoking history, total cholesterol, and LDL-C levels. The results are expressed as hazard ratios (HRs) per 1 μmol/L decrease in α-ketoglutarate with 95% CIs. ROC curves were used to assess prognostic performance. All tests were two-tailed, with *P* < 0.05 considered statistically significant.

## 3 Results

### 3.1 Comparison of oxidative stress indices between the conservative medical treatment group and the control group

Serum oxidative stress markers were compared between the conservative treatment and control groups via independent samples *t* tests with Welch's correction. Compared with the control group, the conservative group presented significantly lower TAC (mean difference: −4.93 kU/L, 95% CI: −5.10 to 4.76), SOD (−13.75 nU/ml, 95% CI: −14.82 to 12.68), CAT (−4.99 U/ml, 95% CI: −5.74 to 4.24), and GSH (−6.16 mg/L, 95% CI: −6.74 to 5.58), as did the control group (6.79 μmol/L, 95% CI: 6.07–7.51) (all *P* < 0.001; Table 2).

**Table 2:** Comparison of oxidative stress markers between the conservative and control groups.

Group	TAC (kU/L)	SOD (nU/ml)	MDA (μmol/L)	CAT (U/ml)	GSH (mg/L)
Conservative (*n* = 159)	10.12 ± 0.52	72.40 ± 3.05	27.84 ± 2.55	22.15 ± 2.66	7.05 ± 1.02
(95% CI)	(10.04–10.20)	(71.92–72.88)	(27.44–28.24)	(21.73–22.57)	(6.89–7.21)
Control (*n* = 59)	15.05 ± 0.61	86.15 ± 3.46	21.05 ± 2.30	27.14 ± 3.02	13.21 ± 2.12
(95% CI)	(14.89–15.21)	(85.25–87.05)	(20.45–21.65)	(26.36–27.92)	(12.66–13.76)
Mean difference (95% CI)	−4.93 (−5.10 to −4.76)	−13.75 (−14.82 to −12.68)	6.79 (6.07 to 7.51)	−4.99 (−5.74 to −4.24)	−6.16 (−6.74 to −5.58)
t (df)	−57.71 (216)	−28.50 (216)	17.92 (216)	−11.86 (216)	−28.81 (216)
*P*	<0.001	<0.001	<0.001	<0.001	<0.001

Data: mean ± SD. CI, confidence interval. Welch's *t* test was used.

### 3.2 Comparison of α-ketoglutarate levels among the three groups

**Figure 1:**
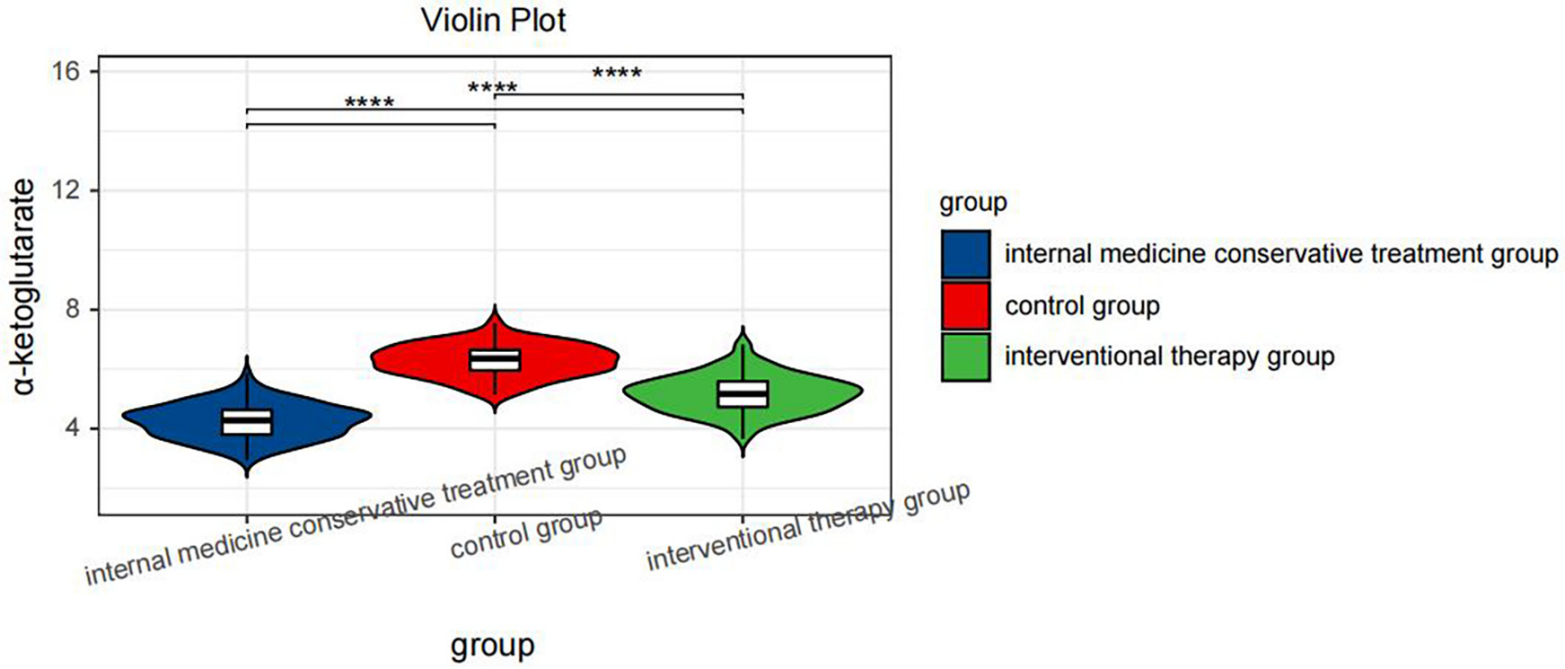
Violin plot comparing the serum α-ketoglutarate levels across hree groups: the internal medicine conservative treatment group, the control group, and the interventional therapy group. Statistical significance is indicated by **** (*P* < 0.0001) between the groups.

**Figure 2:**
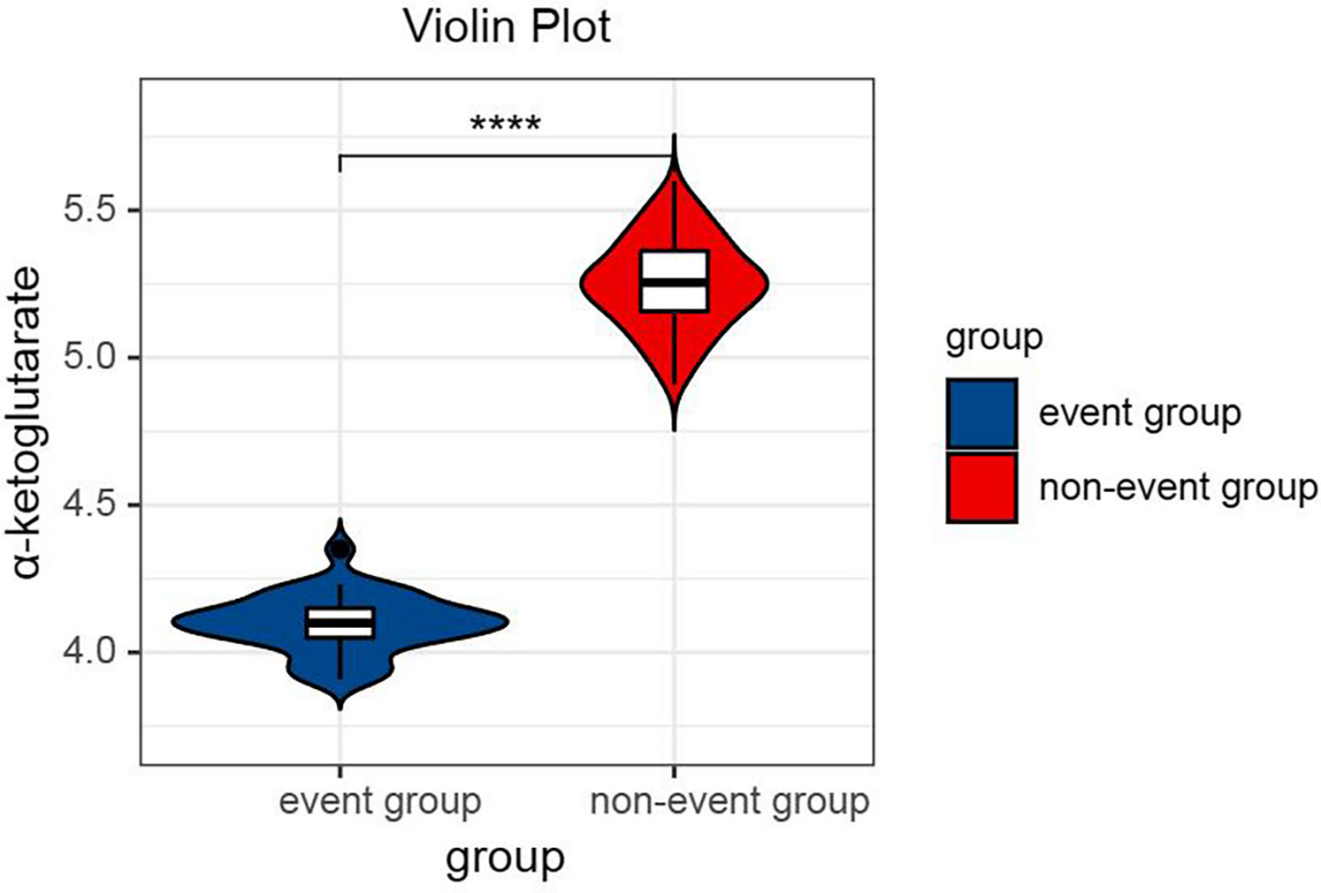
Violin plot comparing the serum α-ketoglutarate levels between the event group and the nonevent group after interventional therapy. Statistical significance is indicated by **** (*P* < 0.0001).

One-way ANOVA with Tukey's *post hoc* test revealed significant differences in α-ketoglutarate levels across the groups [*F*(2,374) = 286.4, *P* < 0.001]. Specifically, the control group presented the highest α-ketoglutarate level (6.28 μmol/L, 95% CI: 6.14–6.42), followed by the interventional treatment group (5.18 μmol/L, 95% CI: 5.08–5.28), and the conservative treatment group presented the lowest level (4.25 μmol/L, 95% CI: 4.16–4.34; all pairwise *P* < 0.001, Table 3), as shown in Figure 1. This trend indicates that α-ketoglutarate levels decrease as the treatment method becomes more aggressive, with the control group exhibiting the highest levels and the conservative treatment group the lowest. ANCOVA adjusted for age, hypertension, and diabetes confirmed that these differences remained significant (*P* < 0.001). In the interventional cohort, α-ketoglutarate levels were significantly lower in the event subgroup than in the nonevent subgroup, with a mean difference of −1.15 μmol/L (95% CI: −1.20 to −1.10; *P* < 0.001, Table 4), as depicted in Figure 2. These findings suggest that lower α-ketoglutarate levels in the event group may be indicative of a greater risk for adverse outcomes postintervention.

**Table 3:** α-Ketoglutarate levels across groups (ANOVA).

Group	*n*	α-ketoglutarate (μmol/L)	95% CI
Conservative	159	4.25 ± 0.58	(4.16–4.34)
Interventional	159	5.18 ± 0.62	(5.08–5.28)
Control	59	6.28 ± 0.53	(6.14–6.42)
F (df1, df2)	286.4 (2, 374)		
*P*	<0.001		

*Post hoc* Tukey test: all pairwise *P* values < 0.001. CI, confidence interval for the mean.

**Table 4:** α-Ketoglutarate in event vs. nonevent subgroups.

Group	*n*	α-ketoglutarate (μmol/L)	95% CI	Mean difference (95% CI)
Event	31	4.10 ± 0.10	(4.06–4.14)	Reference
Nonevent	128	5.25 ± 0.15	(5.22–5.28)	1.15 (1.10–1.20)
t (df)	−40.51 (157)			
*P*	<0.001			

Data: mean ± SD. CI, confidence interval. Independent *t* test.

### 3.3 Correlation analysis between α-ketoglutarate acid and the oxidative stress response in patients with CHD

Significant correlations persisted between α-ketoglutarate and oxidative stress markers after Bonferroni correction (adjusted *α* = 0.01): negative correlations with TAC (*r* = −0.522), SOD (−0.475), CAT (−0.432), and GSH (−0.512) and positive correlations with MDA (*r* = 0.496; all *P* < 0.001; Table 5).

**Table 5:** Correlation between α-ketoglutarate and oxidative stress markers.

Marker	*r*	95% CI	*P*
TAC	−0.522	(−0.602 to −0.432)	<0.001
SOD	−0.475	(−0.561 to −0.381)	<0.001
MDA	0.496	(0.404 to 0.580)	<0.001
CAT	−0.432	(−0.522 to −0.334)	<0.001
GSH	−0.512	(−0.594 to −0.421)	<0.001

Pearson correlation with Bonferroni correction (*α* = 0.01). CI, confidence interval for *r*.

### 3.4 ROC curve of α-ketoglutarate acid

**Figure 3:**
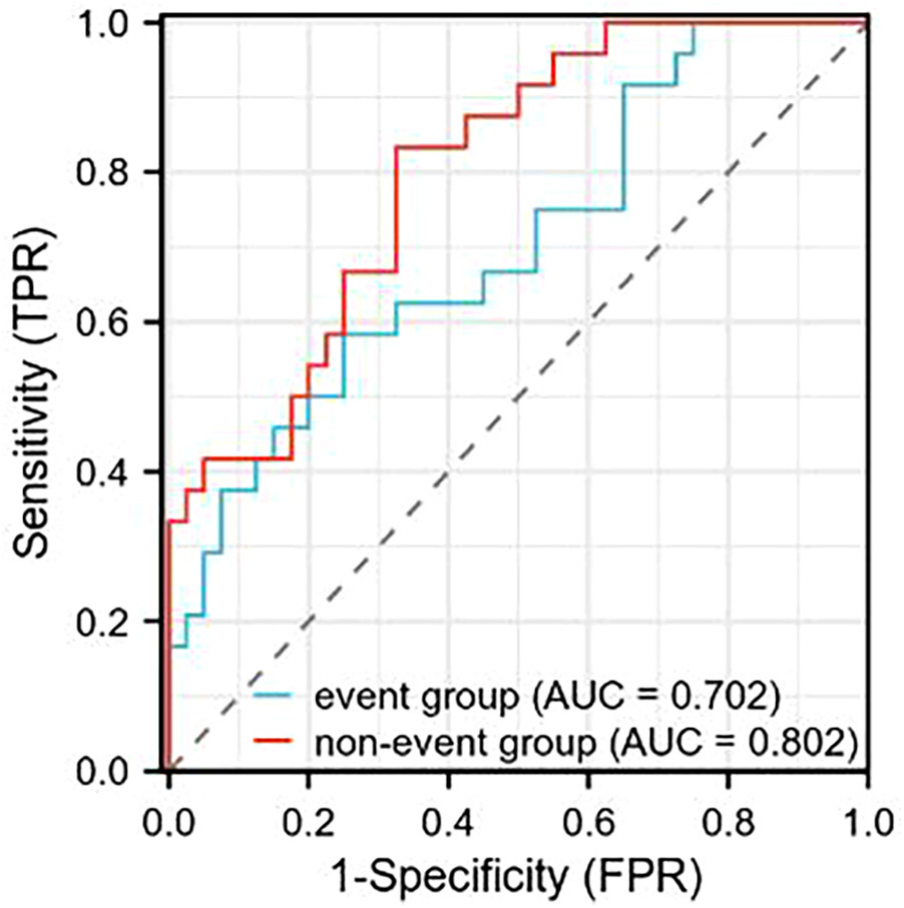
ROC curve analysis of serum α-ketoglutarate levels in predicting primary endpoint events (all-cause death or CHD-related rehospitalization) following interventional therapy. The area under the curve (AUC) for the event group was 0.702, and for the nonevent group, it was 0.802.

After interventional treatment, receiver operating characteristic (ROC) curve analysis was performed to evaluate the prognostic value of serum α-ketoglutarate levels for predicting primary endpoint events (all-cause death or CHD-related rehospitalization). The AUC for α-ketoglutarate was 0.702 (95% CI: 0.603–0.801) in the overall interventional cohort. At the optimal cutoff value of 4.65 μmol/L, α-ketoglutarate demonstrated a sensitivity of 74.2% and specificity of 62.5% for identifying patients at risk of adverse events. Further analysis revealed that the nonevent subgroup had an AUC of 0.802 (95% CI: 0.735–0.869), with increased discriminatory power (sensitivity: 78.1%; specificity: 71.0% at a cutoff of 5.20 μmol/L). The detailed metrics are summarized in Table 6 (Figure 3).

**Table 6:** Prognostic performance of α-ketoglutarate for adverse events after interventional therapy.

Analysis Group	AUC (95% CI)	Cutoff (μmol/L)	Sensitivity (%)	Specificity (%)	PPV (%)	NPV (%)	Youden index
Event prediction^a^	0.702 (0.603–0.801)	4.65	74.2 (23/31)	62.5 (80/128)	34.3	90.2	0.367
Nonevent prediction^b^	0.802 (0.735–0.869)	5.20	78.1 (100/128)	71.0 (22/31)	91.7	46.8	0.491

AUC, area under the curve; CI, confidence interval; PPV, positive predictive value; NPV, negative predictive value.

a Primary endpoint: All-cause death or CHD-related rehospitalization (event group, *n* = 31; nonevent group, *n* = 128).

b Noninvent prediction refers to the ability to identify patients without adverse outcomes.

Optimal cutoffs determined by maximizing the Youden index (sensitivity + specificity − 1).

### 3.5 Multivariable analysis of the prognostic value of α-ketoglutarate

Multivariable Cox regression confirmed that α-ketoglutarate was an independent predictor of primary endpoint events after adjusting for traditional risk factors. In the unadjusted model, each 1 μmol/L decrease in α-ketoglutarate was associated with a 115% increase in event risk (HR = 2.15, 95% CI: 1.58–2.93, *P* < 0.001). After adjustment for age, diabetes, and hypertension (Model 1), α-ketoglutarate remained significantly associated with endpoint events (HR = 1.92, 95% CI: 1.35–2.74, *P* < 0.001). Further adjustment for BMI, smoking status, total cholesterol, and LDL-C (Model 2) maintained this association (HR = 1.87, 95% CI: 1.29–2.71, *P* = 0.001), indicating robust independence from conventional cardiovascular risk factors (Table 7).

**Table 7:** Multivariate Cox regression analysis of α-ketoglutarate for primary endpoint events.

Model	HR (95% CI)	*P* value	Adjustment Variables
Unadjusted	2.15 (1.58–2.93)	<0.001	None
Model 1	1.92 (1.35–2.74)	<0.001	Age, Diabetes, Hypertension
Model 2	1.87 (1.29–2.71)	0.001	Model 1 + BMI, Smoking, Total Cholesterol, LDL-C

HR, hazard ratio per 1 μmol/L decrease in α-ketoglutarate; CI, confidence interval; LDL-C, low-density lipoprotein cholesterol.

## 3 Discussion

In most Western countries, cardiovascular disease accounts for approximately half of total mortality, with CHD ranking first, accounting for more than 50% of vascular disease deaths. More than 80% of sudden cardiac deaths worldwide are caused by CHD, and the remaining 20% are caused by cardiomyopathy, congenital heart disease, ventricular hypertrophy, heart valve disease, and other heart diseases. CHD is caused by arterial stenosis caused by atherosclerosis. Arterial lumen stenosis in patients with CHD is related mainly to the occurrence of atherosclerotic plaques. The accumulation of lipid metabolism factors and increased levels of inflammatory factors aggravate the formation of atherosclerotic plaques. Endothelial dysfunction caused by the formation of atherosclerotic plaques plays a crucial role in the pathogenesis of CHD. In recent years, the incidence of CHD in China has continued to increase, and its high disability and mortality rates have placed a heavy burden on society and families (15). With the continuous development of interventional cardiology, PCI has become widely used to treat CHD, and recent results are obvious (16). However, the prognosis of PCI treatment is affected by factors such as age, sex, and diabetes, hypertension, hyperlipidemia, etc. The prognosis of some patients with CHD is poor, and sudden death may even occur (17).

α-Ketoglutarate is a derivative of glutaric acid with a keto group. It is an important intermediate metabolite in glutamine and tricarboxylic acid cycle metabolism. It can not only generate adenosine triphosphate (ATP) to provide energy for the body and improve vascular endothelial cell function and autophagy but also limit the energy utilization of nutrients in the body by regulating ATP synthesis, thus preventing aging and other related diseases (18, 19). Oxidative stress is caused by an imbalance of antioxidants and free radicals and is an important prerequisite for the occurrence and development of atherosclerosis. Studies have shown that α-ketoglutarate can oxidatively decompose hydrogen peroxide (H_2_O_2_), reduce the production of oxygen free radicals, and prevent lipid peroxidation damage. In addition, by reducing the content of reactive oxygen species generated by cells *in vivo* and *in vitro*, the scavenging rate of oxygen free radicals in the body can be accelerated, thereby increasing their antioxidant capacity and further protecting the vascular endothelium (20). Reports indicate that the level of α-ketoglutarate in the blood of elderly patients with peripheral vascular disease is significantly reduced. α-Ketoglutarate can not only affect the methylation level of vascular endothelial cells but also affect the autophagy and oxidative stress state of vascular endothelial cells, thereby further improving their function (21). The results of this study revealed that, compared with those in the control group, the α-ketoglutarate levels in the intervention group were greater than those in the medical conservative treatment group. The serum TAC, SOD, CAT, and GSH levels in the conservative medical treatment group were lower than those in the control group (*P* < 0.05). The serum MDA level was greater than that in the control group (*P* < 0.05). α-Ketoglutarate is significantly correlated with the oxidative stress response in patients with CHD. Mechanistically, α-ketoglutarate may reduce oxidative stress through multiple pathways: (1) As a direct substrate for glutathione regeneration, it enhances the glutathione antioxidant system to neutralize reactive oxygen species (ROS) and limit lipid peroxidation (22); (2) It activates the AMPK-PGC-1 α/Nrf2 pathway, upregulating endogenous antioxidants (SOD, CAT, HO-1) while suppressing NADPH oxidase-mediated ROS production (23). By restoring redox homeostasis and protecting mitochondrial function, α-ketoglutarate attenuates endothelial dysfunction and atherosclerotic plaque progression, thereby inhibiting CHD development. The observed AKG elevation post-PCI may be attributed to improved oxygen-dependent mitochondrial function. Coronary revascularization restores TCA cycle flux, enhancing α-Ketoglutarate synthesis while alleviating oxidative stress-driven α-Ketoglutarate consumption (24). Critically, multivariable Cox regression analysis confirmed that α-ketoglutarate maintains independent prognostic value after rigorous adjustment for traditional cardiovascular risk factors. Even when accounting for age, diabetes, hypertension, BMI, smoking status, and lipid profiles, each 1 μmol/L decrease in α-ketoglutarate conferred an 87% increased risk of adverse events (HR = 1.87, 95% CI: 1.29–2.71; *P* = 0.001). These findings demonstrate that the predictive capacity of α-ketoglutarate extends beyond established clinical parameters, positioning it as a novel biomarker for risk stratification in interventional cardiology.

Compared to emerging CHD biomarkers, α-ketoglutarate demonstrates competitive prognostic performance. While novel inflammatory markers like growth differentiation factor-15 (GDF-15) achieve AUCs of 0.68–0.72 for major adverse cardiac events (25), and microRNA panels report AUCs up to 0.79 (2), α-ketoglutarate shows comparable discrimination (AUC: 0.80 for non-event prediction). Importantly, α-ketoglutarate outperforms conventional oxidative stress markers like myeloperoxidase [AUC: 0.63–0.67; (26)] in specificity (71.0% vs. 58.2%) while maintaining high sensitivity (78.1%), suggesting unique value in redox metabolism-based risk stratification.

The tricarboxylic acid cycle (TCA) is one of the most basic metabolic processes in the biological world and includes plants, animals, microorganisms, and humans. As a key intermediate metabolite, α-ketoglutarate has attracted increasing attention for its role in health and disease (27). In recent years, studies have shown that α-ketoglutarate can prevent or even reverse atherosclerotic lesions in aged animals. An in-depth study of the relationship between α-ketoglutarate and atherosclerosis will provide new strategies and methods for the prevention and treatment of atherosclerosis (28). This study investigated the relationship between serum α-ketoglutarate levels and the prognosis of CHD patients. The results revealed that the serum α-ketoglutarate level in the event group was lower than that in the nonevent group (*P* < 0.05). According to the ROC curve analysis, among patients undergoing interventional treatment for CHD, α-ketoglutarate had higher sensitivity and specificity in the event-free group. While the AUC of 0.702 for event prediction indicates acceptable discriminatory ability (15), the higher AUC (0.802) observed in the non-event group suggests α-ketoglutarate may be particularly valuable for identifying low-risk patients who could potentially avoid intensive monitoring post-PCI. These findings have value for evaluating the prognosis of patients after CHD interventional treatment.

In summary, α-ketoglutarate can reduce the body's oxidative stress response. The lower the α-ketoglutarate level in the serum is, the greater the likelihood that adverse events will occur. The α-ketoglutarate level has a certain value for evaluating the prognosis of patients after CHD intervention. Proper supplementation with α-ketoglutarate can help reduce oxidative stress in patients with CHD and improve the prognosis of patients undergoing interventional treatment for CHD. However, several limitations should be acknowledged. First, the mechanistic basis of serum α-ketoglutarate regulation remains incompletely characterized. Second, as an observational study, our findings demonstrate an association between α-ketoglutarate and oxidative stress/prognosis but cannot establish causality due to potential residual confounding. Third, the relatively small sample size of the event subgroup (*n* = 31) may have limited the statistical power of the prognostic analyses. Future studies should incorporate longitudinal designs and mechanistic experiments to elucidate causal pathways while extending the follow-up duration in larger cohorts to validate prognostic thresholds.

In summary, α-ketoglutarate demonstrates significant prognostic utility in CHD management. Based on our results, we propose a three-stage clinical application for α-ketoglutarate: (1) pre-procedure risk stratification: Measure baseline levels to identify high-risk patients (AKG <4.65 μmol/L) warranting intensified peri-procedural care; (2) post-PCI triage: Utilize AKG ≥5.20 μmol/L to identify low-risk candidates suitable for early discharge and reduced monitoring; (3) long-term management: Serial AKG measurements during follow-up could guide secondary prevention intensity, with persistently low levels prompting advanced therapies. This approach aligns with precision medicine paradigms for cardiac risk biomarkers, though protocol standardization and cost-effectiveness studies remain needed.

## Data Availability

The original contributions presented in the study are included in the article/Supplementary Material, further inquiries can be directed to the corresponding author.
